# Individualized or Uniform De-Escalation Strategies for Antiplatelet Therapy in Acute Coronary Syndrome: A Review of Clinical Trials with Platelet Function Testing and Genetic Testing-Based Protocols

**DOI:** 10.3390/ijms24109071

**Published:** 2023-05-22

**Authors:** Oumaima El Alaoui El Abdallaoui, Dániel Tornyos, Réka Lukács, Dóra Szabó, András Komócsi

**Affiliations:** 1 Doctoral School of Health Sciences, Faculty of Health Sciences, University of Pécs, 7621 Pécs, Hungary; oumaimaelalaouielabdallaoui@gmail.com; 2 Department of Interventional Cardiology, Heart Institute, Medical School, University of Pécs, 7624 Pécs, Hungary; tornyosdaniel@gmail.com (D.T.); lukacs.reka@pte.hu (R.L.); szabo.dora@pte.hu (D.S.)

**Keywords:** antiplatelet therapy, de-escalation, acute coronary syndrome, platelet function testing, genetic testing, individualized therapy

## Abstract

This comprehensive literature review assessed the effectiveness of precision medicine approaches in individualizing P2Y12 de-escalation strategies, such as platelet function testing guidance, genetic testing guidance, and uniform de-escalation, for acute coronary syndrome (ACS) patients undergoing percutaneous coronary intervention (PCI). Analyzing six trials with a total of 13,729 patients, the cumulative analyses demonstrated a significant reduction in major adverse cardiac events (MACE), net adverse clinical events (NACE), and major and minor bleeding events with P2Y12 de-escalation. Specifically, the analysis found a 24% reduction of MACE and a 22% reduction of adverse event risk (relative risk (RR) 0.76, 95% confidence interval (CI): 0.71–0.82, and RR: 0.78, 95% CI 0.67–0.92, respectively). Reductions in bleeding events were highest with uniform unguided de-escalation, followed by guided de-escalations, while ischemic event rates were similarly lower across all three strategies. Although the review highlights the potential of individualized P2Y12 de-escalation strategies to offer a safer alternative to the long-term potent P2Y12 inhibitor-based dual antiplatelet therapy, it also indicates that laboratory-guided precision medicine approaches may not yet offer the expected benefits, necessitating further research to optimize individualized strategies and evaluate the potential of precision medicine approaches in this context.

## 1. Introduction

Acute coronary syndrome (ACS) encompasses a spectrum of conditions characterized by a sudden decrease in blood flow to the heart, which can be life-threatening and necessitate prompt medical intervention to restore blood supply, prevent myocardial damage, and address potential complications such as ischemia and arrhythmia [1]. Antiplatelet therapy is a critical component in the management of ACS, as it inhibits the formation and progression of blood clots that may obstruct coronary arteries. For most cases of ACS, mechanical reperfusion through balloon dilation and stent implantation in the affected coronary arteries is the preferred treatment approach, with antiplatelet therapy playing a key role in preventing thrombosis at the intervention site [2].

Nonetheless, antiplatelet therapy carries some risks, with bleeding complications and MACE occurring in up to 5% and 5.8% of patients, respectively [3,4]. Consequently, it is vital to strike a balance between the benefits and risks of antiplatelet therapy in ACS patients. In recent years, several de-escalation strategies involving platelet function testing (PFT) and genetic testing-based protocols have been developed to minimize bleeding risk while preserving the effectiveness of antiplatelet therapy. This article reviews the current evidence on individualized or uniform de-escalation strategies for antiplatelet therapy in ACS patients, with an emphasis on the role of PFT and genetic testing-based protocols in informing treatment decisions.

## 2. Pathophysiological Background

Platelets play a vital role in hemostasis. They become activated upon encountering damaged blood vessels or tissues. Various mechanisms can initiate platelet activation, including pathways mediated by thrombin, collagen, and adenosine diphosphate (ADP) [5].

The ADP-mediated mechanism is one of the most crucial pathways for platelet activation. ADP binds to P2Y1 and P2Y12 receptors on platelet surfaces, activating intracellular signaling pathways that cause platelets to change shape, secrete granules, and aggregate [6].

The P2Y1 receptor is responsible for inducing rapid calcium influx into the platelet, leading to shape change and granule secretion after it is linked to Gαq. The P2Y12 receptor is involved in platelet aggregation by activating the integrin alpha IIb beta 3 on the platelet surface and completing the ADP-dependent platelet aggregation response initiated by P2Y1 as well as the ADP-dependent amplification of platelet aggregation induced by other agents such as Gq-coupled serotonin receptors, Gq and G12/13-coupled TXA2 and PAR-1 receptors, immune complexes, or when platelets are activated by collagen through the GPVI/tyrosine kinase/PLCγ2 pathway. This process results in the cross-linking of adjacent platelets and the formation of a platelet plug to seal the site of injury [7].

Platelet activation is a complex process that involves other agonists such as thrombin, thromboxane, and collagen. Targeting platelet activation with antiplatelet therapy can help prevent platelet aggregation and the formation of blood clots that may lead to heart attacks and strokes. Combining antiplatelet treatments that block multiple signaling pathways, such as aspirin and a P2Y12 inhibitor, is often used in high-risk patients, including those with ACS and following coronary intervention. Additionally, protease-activated receptor-1 (PAR-1) inhibition has been investigated as an alternative treatment option [8]. Vorapaxar, a PAR-1 inhibitor, has been a significant focus of drug development. Studies involving these drugs have demonstrated some success; however, concerns about increased bleeding risk have overshadowed their positive results [9,10].

Clopidogrel was the primary P2Y12 receptor antagonist in clinical practice for many years, but its use exhibited drawbacks such as delayed onset of action, high interindividual response variability, and high residual platelet reactivity during treatment. This was associated with an increase in ischemic events such as stent thrombosis, primarily among high-risk patients with ACS [11].

Prasugrel and ticagrelor represent the next generation of ADP receptor antagonists with a shorter onset of action and more consistent inhibition of platelet aggregation. They have demonstrated a higher risk reduction for thrombosis compared to clopidogrel in patients with ACS in the TRITON-TIMI 38 and PLATO trials [12,13]. However, trials testing these drugs in lower-risk populations failed to prove their benefit compared to clopidogrel. Notably, while the benefits of more potent antiplatelet therapy are more pronounced during the earliest weeks after intervention, bleeding events accumulate during long-term antiplatelet treatment. As both ischemic and bleeding events pose significant prognostic risks for patients with ACS, recent trials have sought to personalize antiplatelet therapy based on these characteristics, adjusting antiplatelet use according to changes in risk during the clinical course.

## 3. Role of Platelet Function Testing in Assessing P2Y12 Inhibitor Therapy

PFT is a valuable ex vivo method for evaluating the effectiveness of clopidogrel treatment [14]. Clopidogrel, a prodrug, requires a two-step activation process in the liver to produce its active metabolite. The absorbed clopidogrel competes with other substrates for the limited metabolic capacity of the liver enzyme CYP2C19 and is subject to non-specific inactivation by plasma esterases. Genetic variations in CYP2C19 activity and the esterase-mediated degradation of over 60% of the drug, as well as absorption issues in critically ill patients, can lead to insufficient active metabolite production and an inadequate response to clopidogrel, increasing the risk of blood clots [11]. ADP-specific PFTs are designed to detect alterations in P2Y12-specific signaling or aggregation and may be used to monitor the achieved antiplatelet action.

Various methods exist for PFT, including light transmission aggregometry (LTA), VerifyNow P2Y12 assay, and Multiplate analyzer. LTA, considered the most reliable method, is time-consuming and requires specialized equipment. The VerifyNow P2Y12 assay and Multiplate analyzer are faster point-of-care methods, but they have limitations in sensitivity and specificity [15].

If patients exhibit a poor response to clopidogrel, alternative antiplatelet medications such as ticagrelor or prasugrel may be more effective. PFT can also be used to monitor the effectiveness of these alternative therapies and adjust dosages as necessary [16].

The limitations of these PFT methods have been discussed extensively elsewhere [17]. PFT analyses were included in trials aiming to characterize optimal antiplatelet dosages. They are considered helpful in identifying individuals with a poor treatment response and can aid in selecting appropriate alternative treatments.

## 4. Genetic Background of Interindividual Response Variability by Clopidogrel

Genetic polymorphisms impacting the function of enzymes responsible for their metabolism can lead to variable levels of clopidogrel metabolism and platelet inhibition, potentially affecting clinical outcomes [18].

Several cytochrome P450 (CYP) enzymes, including CYP2C19, are involved in clopidogrel metabolism. Genetic polymorphisms affecting CYP2C19 function can result in variable levels of clopidogrel metabolism and platelet inhibition, ultimately impacting clinical outcomes [18].

The most common CYP2C19 variant alleles are the loss-of-function alleles *2 and *3, which result in reduced enzymatic activity and lower levels of active metabolite formation. In contrast, the gain-of-function allele *17 is associated with increased enzymatic activity and higher levels of active metabolite formation. Studies have shown that carriers of CYP2C19 loss-of-function alleles have a higher risk of recurrent ischemic events and stent thrombosis compared to non-carriers, particularly in patients with ACS undergoing PCI. This is likely due to decreased platelet inhibition and a lower antiplatelet effect of clopidogrel in these patients [19].

**Figure 1:**
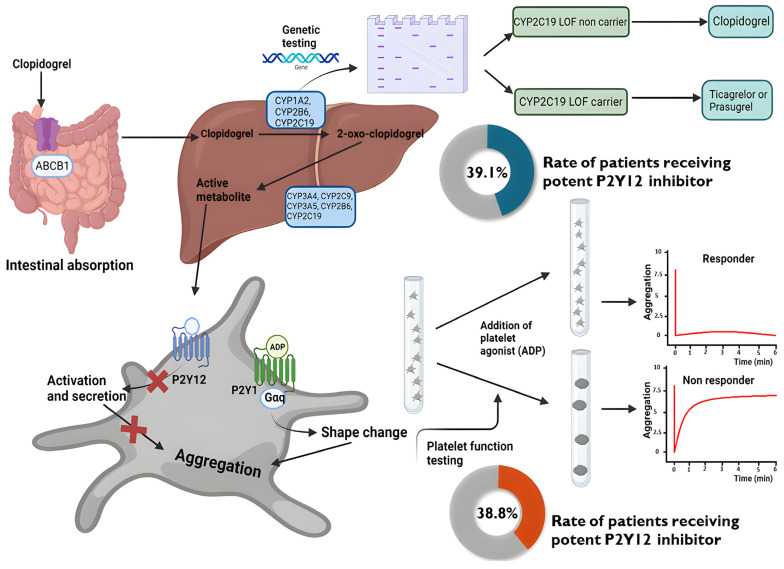
The metabolism of clopidogrel in the liver is genetically determined by the CYP2C19 enzyme. Genetic carrier status and the in vitro measurement of residual platelet function testing (PFT) may be used to identify patients with a higher risk for clopidogrel inefficacy. P2Y12 de-escalation trials using PFT and genetic testing-guided trials maintained long-term potent P2Y12 inhibitor treatment in the identified high-risk subset (rates in orange and blue, respectively). (Created with BioRender.com accessed on 21 April 2023).

In addition to CYP2C19, other genetic polymorphisms affecting clopidogrel metabolism have been studied, such as ABCB1 and PON1, but their clinical relevance remains unclear. Rideg et al. studied the effect of various SNPs, such as Cytochrome 2C19 (CYP2C19) loss-of-function (LOF; *2, *3) and gain-of-function (GOF; *17) allelic variants, along with ABCB1 (3435 C→T and 2677 G→T/A) and paraoxonase-1 (PON-1; 192 Q→R), on post-clopidogrel platelet reactivity and clinical outcome. They found that genetic variants in CYP2C19 had a gene-dose effect on post-clopidogrel platelet reactivity, but neither ABCB1 nor PON-1 genotypes significantly influenced platelet reactivity or outcome [19] (Figure 1).

The clinical significance of genetic testing for CYP2C19 polymorphisms is still under debate. The 2017 American College of Cardiology/American Heart Association guidelines recommend testing for CYP2C19 loss-of-function alleles in patients undergoing PCI who will receive clopidogrel therapy [20]. However, other guidelines, such as those from the European Society of Cardiology, do not recommend routine genetic testing due to the lack of conclusive evidence regarding its clinical utility. The optimal approach to genetic testing and its clinical usefulness remains to be determined.

Numerous studies have been conducted to personalize antiplatelet therapy for patients undergoing percutaneous coronary intervention (PCI). The GRAVITAS trial showed that high on-treatment platelet reactivity (HTPR), evaluated by assays such as LTA, VerifyNow, Multiplate, or VASP, is a strong marker for worse outcomes in patients after PCI [21]. However, HTPR is not the only determinant of clinical outcomes, as other clinical and procedural factors also play a role. In the POPULAR study, adding HTPR to traditional risk factors only modestly improved the overall predictive value of the model in elective patients after PCI. Nonetheless, platelet function monitoring may be useful in combining the prognostic impact of a patient’s fixed clinical makeup with a potentially corrigible estimate of a drug’s effect.

The ARCTIC-GENE study aimed to adjust antiplatelet therapy according to CYP2C19 genotypes, clopidogrel pharmacodynamic response, and assessed clinical outcomes in patients who underwent stent implantation. The study included 1394 patients who were genotyped for loss- and gain-of-function CYP2C19 alleles and randomized to a strategy of platelet function monitoring with drug adjustment or a conventional strategy without monitoring and drug adjustment. The study found that slow metabolizers, identified as carriers of at least one loss-of-function allele CYP2C19*2, were more likely to be poor responders to antiplatelet therapy at randomization and 14 days later. However, the study did not find any significant difference in the primary study outcome, defined as the composite of death, myocardial infarction, stent thrombosis, stroke, or urgent revascularization 1 year after stent implantation, between slow and rapid metabolizers. The study concluded that the genetic clopidogrel profile was a good marker of platelet function response but added little to the pharmacodynamic information used in the study to adjust antiplatelet therapy [22].

The POPular Genetics trial also failed to show a significant reduction in clinical endpoints with the use of genetic testing-based individualized antiplatelet strategy. The study randomized 2488 ACS patients to either standard DAPT with aspirin and clopidogrel or to CYP2C19 genotyping guided treatment. The latter group received either clopidogrel or ticagrelor based on CYP2C19 genotype. The study found no significant difference between the two groups in terms of the composite endpoint of death from cardiovascular causes, myocardial infarction, stroke (5.1% vs. 5.9%, HR: 0.89, [95% CI: 2.0–0.7]), or major bleeding at 12 months (9.8% vs. 12.5%, HR: 0.78, [95% CI: 0.61–0.98]) [23].

## 5. The Use of P2Y12 Inhibitors in Acute Coronary Syndrome

Coronary artery disease (CAD) is a prevalent and severe medical condition that leads to significant morbidity and mortality worldwide. ACS typically results from plaque rupture or erosion, leading to blood clot formation. PCI with stent placement is a common treatment for ACS patients. Antiplatelet medications, particularly P2Y12 inhibitors, play a crucial role in reducing the risk of recurrent ischemic events in patients undergoing PCI, but they may also increase the likelihood of bleeding [24].

Prasugrel, a third-generation thienopyridine, irreversibly inhibits the P2Y12 receptor. The TRITON-TIMI 38 trial compared prasugrel to clopidogrel in patients with ACS undergoing PCI. Prasugrel reduced the risk of the primary endpoint—a composite of death from cardiovascular causes, nonfatal myocardial infarction, or nonfatal stroke—compared to clopidogrel (9.9% vs. 12.1%, HR: 0.81, [95% CI: 0.73–0.90]). However, prasugrel was associated with an increased risk of major bleeding (2.4% vs. 1.8%, HR: 1.32, [95% CI: 1.03–1.68]), including fatal bleeding (0.4% vs. 0.1%, HR: 3.39, [95% CI: 1.78–6.45]) [12].

Ticagrelor is a reversible P2Y12 inhibitor with a faster onset of action than clopidogrel and does not require hepatic metabolism for activation. The PLATO trial investigated ticagrelor compared to clopidogrel in ACS patients. There was no difference in terms of the risk of the primary endpoint—PLATO major bleeding—between ticagrelor and clopidogrel treated patients (11.6 vs. 11.2%, *p* = 0.43). Ticagrelor reduced the risk of the primary endpoint—a composite of death from cardiovascular causes, myocardial infarction, stroke, and bleeding—compared to clopidogrel (7.86 vs. 8.97%, HR: 0.87, [95% CI 0.77–0.98], *p* = 0.026). However, it was associated with an increased non-CABG major bleeding (4.5 vs. 3.8%, *p* = 0.02) and non-procedure related major bleeding (3.1 vs. 2.3%, *p* = 0.05). The risk of fatal bleeding was similar between the two groups (0.3 vs. 0.3%, *p* = 0.66) [13].

The ISAR-REACT 5 trial conducted a head-to-head comparison of the two potent P2Y12 inhibitors. In this trial, the composite endpoint of cardiovascular death, myocardial infarction, or stroke showed a highly significant reduction favoring prasugrel vs. ticagrelor (HR: 1.36, [95% CI: 1.09–1.70], *p* = 0.006), and bleeding events did not differ between groups (HR: 1.12, [95% CI: 0.83–1.51], *p* = 0.45) [25].

In conclusion of all these trials, both prasugrel and ticagrelor have been shown to minimize the risk of recurrent ischemic events in ACS patients undergoing PCI compared to clopidogrel. The ISAR-REACT 5 trial directly compared prasugrel and ticagrelor, demonstrating that prasugrel was associated with a lower risk of the primary endpoint, which included death, myocardial infarction, or stroke, compared to ticagrelor. However, the risk of bleeding (major bleeding events defined by the Bleeding Academic Research Consortium (BARC) type 3 or 5) was not significantly different between the two groups. Therefore, while prasugrel showed superior efficacy compared to ticagrelor, the risk of bleeding between the two drugs was comparable.

## 6. Genetic Testing-Based P2Y12 De-Escalation Strategy

**Figure 2:**
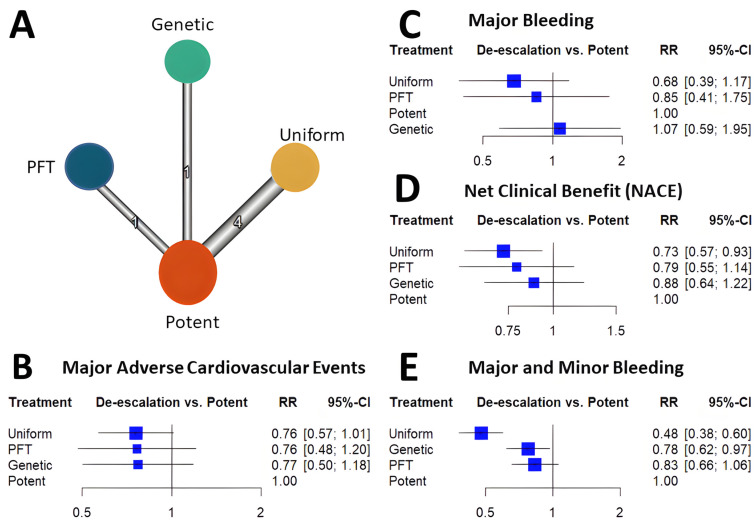
Network meta-analysis results of randomized trials of P2Y12 de-escalation. Network graph depicts the available trial information. Nodes are proportional with the number of patients included and edges are proportional with the number of studies performed (Panel (**A**)). Forest plots depict the results of network meta-analysis showing the risk ratio (RR) and its 95% confidence interval (95% CI) compared to the control arm using long-term potent P2Y12 inhibition. Major adverse cardiovascular events (MACE) are defined as composites of cardiovascular mortality, myocardial infarction, and stroke. Net clinical benefit (NACE) is defined as composite of MACE and major bleeding (Panel (**B**–**E**)).

Genetic testing can help identify individuals who may not respond well to clopidogrel, which could have long-term implications for their ischemic risk. However, selective use of potent P2Y12 inhibitors in loss-of-function carriers did not result in an improvement of clinical outcomes [21,22]. The TAILOR-PCI trial tested a carrier status-based de-escalation strategy. This trial randomized 5302 ACS patients undergoing PCI to either standard dual antiplatelet therapy (DAPT) with aspirin and clopidogrel or a genotype-guided strategy in which CYP2C19 genotyping was used to determine the choice of P2Y12 inhibitor. The study found that genotype-guided therapy was non-inferior to standard DAPT in terms of the primary endpoint of cardiovascular death, myocardial infarction, stroke, stent thrombosis, or severe bleeding at 12 months (4.0% vs. 5.9%, HR: 0.66, [95% CI: 0.43–1.02], *p* = 0.06) [26] (Table 1). Both the rate of major adverse cardiovascular events (MACE) and the net clinical benefit showed a beneficial trend in this trial; however, the expected lower rate of major bleeding was not reflected in the trial results (Figure 2).

## 7. Platelet Function Testing-Based P2Y12 De-Escalation Strategy

A randomized clinical trial investigated the feasibility and safety of a PFT-based P2Y12 de-escalation strategy in ACS patients. The study aimed to assess whether using PFT to guide P2Y12 inhibitor de-escalation could reduce bleeding complications while maintaining adequate platelet inhibition.

The TROPICAL-ACS trial randomized 2610 ACS patients undergoing PCI to either standard DAPT with aspirin and prasugrel or a de-escalation strategy guided by PFT. In the de-escalation arm, patients received prasugrel for one week followed by clopidogrel for another week. Long-term P2Y12 inhibitor treatment was determined based on the results of the ADP-specific platelet function assay. Patients with acceptable residual platelet reactivity continued clopidogrel, while those with high reactivity were switched back to prasugrel. The latter group constituted 38.8% of the de-escalation arm. The study found that PFT-guided de-escalation was non-inferior to standard DAPT with regard to the composite endpoint of death, myocardial infarction, stroke, and bleeding at 1 year (7% vs. 9%, *p* = 0.0004 for non-inferiority, HR: 0.81, [95% CI: 0.62–1.06], *p*-superiority = 0.12) [27].

Similar to genetic testing, the rates of MACE and net clinical events showed beneficial trends, and a 15% reduction in major bleeding risk was also observed. However, none of these reached the level of statistical significance (Figure 2).

## 8. Trials with Uniform P2Y12 De-Escalation Strategy

Several trials have investigated de-escalation protocols for P2Y12 treatment without considering patient-specific genetic or platelet function data. These trials compared long-term, potent DAPT to protocols that switched patients from potent inhibitors to clopidogrel after a predetermined period.

The TOPIC trial (testing responsiveness to platelet inhibition on chronic antiplatelet treatment for acute coronary syndromes) randomized 646 ACS patients on DAPT to either switch to clopidogrel or continue the newer P2Y12 inhibitor one month after PCI. The primary endpoint of cardiovascular death, myocardial infarction, stroke, or stent thrombosis occurred in 26.3% of patients in the unswitched group and in 13.4% of the switched group (HR: 0.48, [95% CI: 0.34–0.68], *p* < 0.01). No significant difference in ischemic endpoints was reported between the two groups, while bleeding occurred in 4.0% of patients in the switched DAPT and 14.9% in the unswitched DAPT group (HR: 0.30, [95% CI: 0.18–0.50], *p* < 0.01) [28].

The HOST-REDUCE-POLYTECH-ACS trial randomized 2338 ACS patients on DAPT to either continue their current P2Y12 inhibitor dose of prasugrel (10 mg) or receive a lower dose of prasugrel (5 mg). The primary endpoint of a composite of cardiovascular death, myocardial infarction, definite stent thrombosis, or ischemic stroke occurred in 7.2% of patients in the de-escalation group and 10.1% of patients in the standard care group (*p*-non-inferiority < 0.0001, HR: 0.70, [95% CI: 0.52–0.92], *p*-equivalence = 0.012). There was no increase in ischemic risk in the de-escalation group compared with the conventional group (HR: 0.76, [95% CI: 0.40–1.45], *p* = 0.40), and the risk of bleeding events was significantly decreased (HR: 0.48, [95% CI: 0.32–0.73], *p* = 0.0007) [29].

The TALOS-AMI trial randomized 2697 patients on DAPT to either undergo de-escalation to clopidogrel with aspirin or continue DAPT with ticagrelor. The primary endpoint of net adverse clinical events (NACE), including cardiovascular death, myocardial infarction, stroke, and BARC 3 or 5 bleeding, occurred in 4.7% of patients in the de-escalation group and 8.3% of patients in the control group (HR: 0.58, [95% CI: 0.38–0.87], *p* = 0.009), with a significant decrease in bleeding (HR: 0.52, [95% CI: 0.35–0.77], *p* = 0.001) and no increase in ischemic events [30].

Ueno et al. randomized 136 ACS patients on DAPT to either undergo de-escalation to clopidogrel with aspirin or continue DAPT with prasugrel. The primary endpoint was the mean P2Y12 reaction unit (PRU) at week 6, which was significantly lower in the continued group relative to the switched group (140.7 and 183.0, respectively; *p* = 0.001) [31].

## 9. Comparison of Approaches

Comparing the effectiveness of the three de-escalation approaches to P2Y12 de-escalation, including PFT guidance, genetic testing guidance, and uniform de-escalation without laboratory guidance, is challenging due to variations in patient populations, follow-up durations, and endpoints among the trials. Notably, none of these individual trials found a significant reduction in major bleeding, MACE, or net clinical benefit. However, their results supported that protection against ischemic events is not compromised with de-escalation compared to long-term potent P2Y12 treatment.

The risk and benefit of de-escalation related to other antiplatelet strategies were assessed in multiple recent analyses. A recent network meta-analysis aimed to compare the efficacy and safety of different approaches linking standard long-term DAPT with potent P2Y12 antagonists to strategies based on earlier aspirin cessation and potent P2Y12 inhibitor monotherapy after coronary intervention [32]. Ten randomized controlled trials with a total of 42,511 participants were included. They compared four different strategies for abating DAPT: PFT-based P2Y12 de-escalation, genetic testing-based P2Y12 de-escalation, uniform unguided P2Y12 de-escalation, and P2Y12 monotherapy, including ticagrelor monotherapy and clopidogrel arms, which allowed a broader context to relate the efficacy and safety of abatement strategies.

The authors found that both P2Y12 inhibitor de-escalation and P2Y12 inhibitor monotherapy reduce ischemic events and all bleeding (including major and minor events) among PCI-treated ACS patients. However, the different severity of bleeding was differently affected by the abatement strategies. With ticagrelor monotherapy, both major and minor bleeding event risk was significantly reduced, while with de-escalation, only the risk of minor bleeding was significantly reduced.

**Figure 3:**
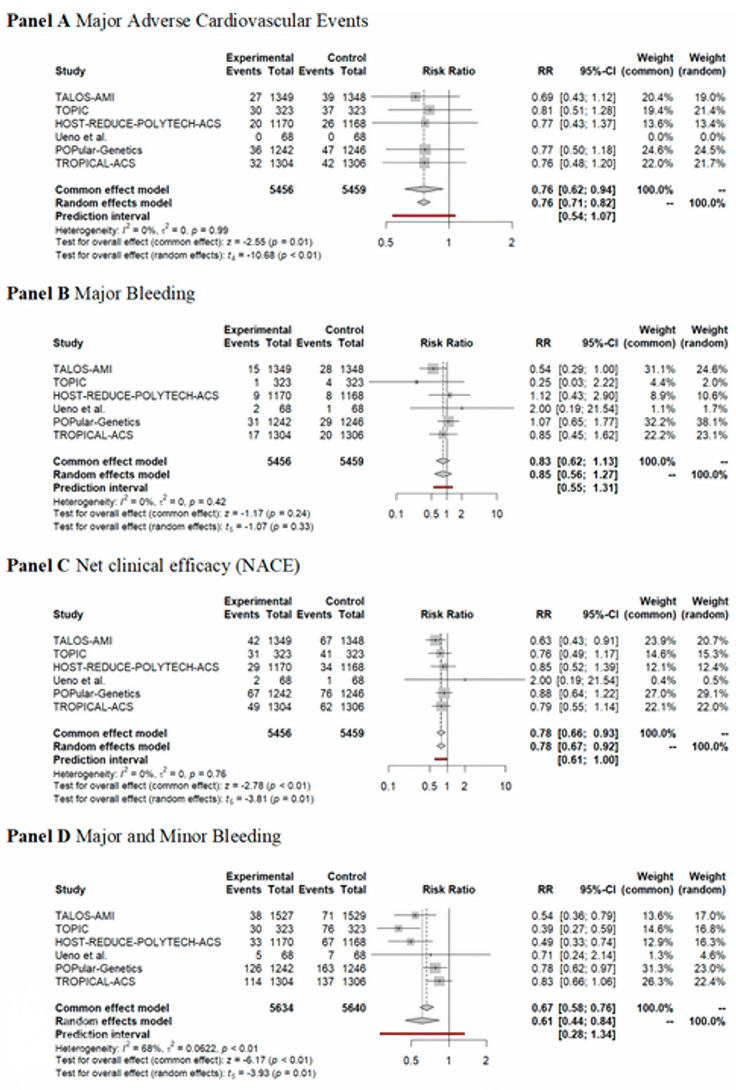
Forest plots depicting clinical endpoints of P2Y12 de-escalation strategies. Panels depict the relative risk of MACE (Panel (**A**)), major bleeding (Panel (**B**)), NACE (Panel (**C**)), and all bleeding defined as major and minor bleeding events (Panel (**D**). (source of data: [32]).

**Figure 4:**
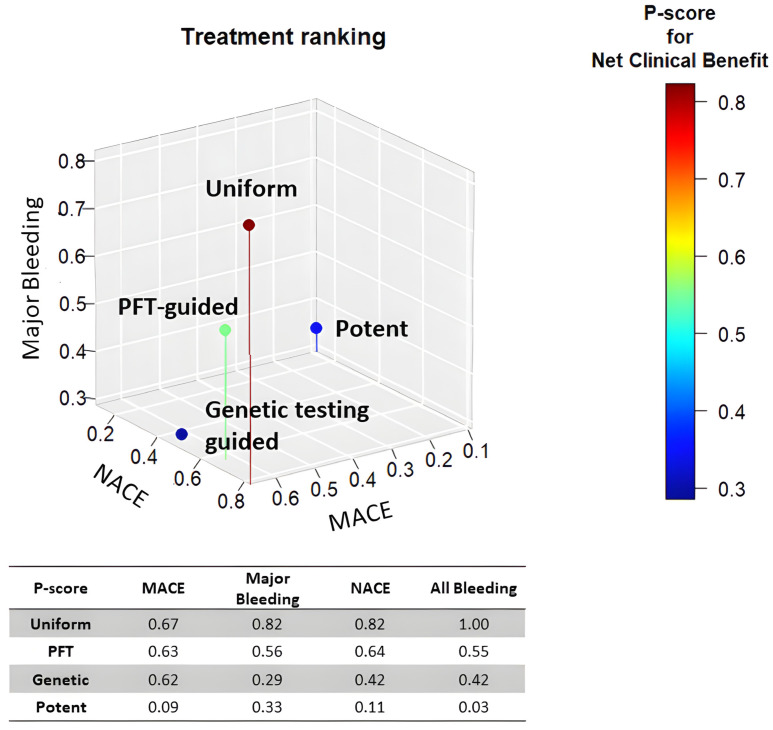
Treatment ranking of P2Y12 de-escalation strategies. The scatterplot depicts the treatment ranking with regard to the risk of MACE, major bleeding, and NACE. Uniform de-escalation was ranked first in all analyses.

Among the de-escalation strategies, uniform de-escalation exhibited the highest reduction in bleeding, followed by genetic testing-guided de-escalation, while PFT-guided de-escalation did not show any significant reduction in bleeding (Figure 2). These trends reached significant levels for all bleeding and minor bleeding, but regarding major bleeding, none of the individual de-escalation strategies or the cumulative estimate of the de-escalation trials reflected a significant reduction (Figure 3). While results of the bleeding risk reduction remained behind expectations for de-escalation strategies, an unexpected benefit was unveiled. Contrary to the anticipated trade-off of accepting a certain increase in ischemic risk, all three P2Y12 inhibitor de-escalation strategies resulted in a similarly lower rate of ischemic events (Figure 4). As these trials were not powered to assess individual endpoints, the cumulative analysis of more than 10,000 randomized patients reflected a highly significant 24% reduction of MACE without signs of major heterogeneity among the trials. Similarly, in net clinical benefit analyses, a significant 22% reduction of adverse event risk was found (Figure 3).

An extensive network meta-analysis conducted by Kuno et al. aimed to assess the efficacy and safety of various dual antiplatelet therapy (DAPT) approaches. The employment of broader inclusion criteria permitted a higher number of trials with less stringent requirements regarding de-escalation. The analysis incorporated 19 randomized controlled trials, totaling 69,746 patients, and evaluated six distinct DAPT strategies, including aspirin and clopidogrel, aspirin and low-dose prasugrel, aspirin and standard-dose prasugrel, aspirin and ticagrelor, as well as an unguided de-escalation strategy and guided selection strategy. Although this approach may facilitate a better understanding of de-escalation within a broader range of therapeutic options, it also carries the risk of network results being influenced or dominated by indirect comparisons. Results of Kuno et al.’s findings were in agreement, indicating that unguided de-escalation was associated with a reduced risk of major adverse cardiovascular events (MACE) when compared to DAPT regimens [33]. Our further analyses revealed no significant difference in MACE risk between guided and unguided strategies, but all studies demonstrated similar reductions that reached statistical significance due to the larger cumulative number of patients included in unguided de-escalation trials.

While ischemic event outcomes suggested a similar benefit for de-escalation with or without laboratory guidance, bleeding rates presented a more heterogeneous picture. A key distinction between our analysis and that of Kuno et al. is that the latter grouped the TROPICAL-ACS and POPULAR-GENETIC trials in the same category. The notable increase in major bleeding in the latter trial, despite significant reductions in major and minor bleeding with genetic testing-based de-escalation, remains unexplained. We believe this discrepancy justifies not grouping these two trials together.

It is essential to note that none of the trials were designed to demonstrate differences in MACE or major bleeding, but rather to establish non-inferiority based on composite endpoints. Complementing Kuno et al.’s analysis, we demonstrated a significant improvement in net clinical benefit with de-escalation strategies. We concur that both guided de-escalation approaches resulted in a higher number of prasugrel treatments in the de-escalation arm, which may explain the less pronounced reduction in major and minor bleeding rates. This observation, combined with cost and logistical concerns, renders unguided de-escalation a more attractive option [33].

We lack a clear mechanistic explanation for the risk reduction, but together with the findings of minor bleeding rate, it has been hypothesized that reduction of these nuisance events may have permitted a more tolerable treatment with higher compliance. If this hypothetical higher adherence translated to the observed clinical benefit, however, we lack conclusive data [32]. The rate of bleeding events may also be influenced by additional factors. Both genetic testing and PFT were incorporated into the de-escalation strategies to implement pharmacokinetic-based risk stratification for identifying patients at the highest risk. However, the practical application of this strategy led to approximately 40% of patients in the individualized treatment arm receiving clopidogrel. A selection strategy resulting in a higher rate of potent treatment might be the reason why these trials’ observed bleeding risk reduction fell short of expectations. It has been suggested that platelet function measurements’ negative predictive values are excellent, potentially providing a valuable tool for identifying patients who can safely remain on clopidogrel therapy. For instance, in a group of ACS patients with access to more potent antiplatelet drugs, continuing clopidogrel therapy may be non-inferior to switching to prasugrel or ticagrelor. However, the positive predictive values of platelet function measurements are mostly fair or poor. While platelet function tests assess residual platelet reactivity, the connection between ischemic risk and genetic predisposition may be even weaker, which could explain the discrepancies in these trials.

Cumulative analyses of P2Y12 inhibitor de-escalation studies demonstrated significant benefits in MACE, NACE, and major + minor bleeding, with slightly greater benefits observed in the uniform studies. However, major bleeding did not show a significant reduction; it was more prevalent in uniform studies, followed by PFT de-escalation strategies, and lastly, genetic testing de-escalation, which exhibited a lesser trend of major bleeding reduction. These results might be attributed to the long-term prasugrel treatment in the de-escalation arms (40%) of both PFT and genetic testing de-escalation, which can impact clinical outcomes, particularly bleeding events. Additionally, these results suggest that risk assessment with PFT may be more precise compared to metabolizer status. Nonetheless, further studies will be necessary to support these assumptions (Figure 3).

Most trials demonstrated trends for improvement concerning these endpoints. A cumulative analysis resulted in a significant reduction in all three endpoints (Figure 2 and Figure 3).

In summary, network analyses suggest that uniform unguided de-escalation may be an effective strategy for reducing potent P2Y12 antagonist-based DAPT after coronary intervention (Figure 4). However, this approach might be associated with an increased risk of ischemic events, as it does not consider each patient’s individual bleeding and ischemic risk to select the optimal approach for DAPT abatement.

Overall, these network analyses suggest that uniform unguided de-escalation may be an effective strategy for abating potent P2Y12 antagonist-based DAPT after coronary intervention (Figure 4). However, this approach may be associated with an increased risk of ischemic events since it does not take into consideration the individual patient’s bleeding and ischemic risk in order to select the optimal approach for DAPT abatement.

In conclusion, uniform unguided P2Y12 de-escalation strategies have consistently shown a reduction in bleeding events without compromising efficacy. Genetic testing-guided de-escalation strategies and de-escalation using PFT guidance provided results showing no difference in bleeding or ischemic events between the de-escalation group and the standard group (4.0% vs. 5.9% and 7% vs. 9%, respectively). Overall, the use of uniform unguided de-escalation appears to be the most effective strategy in reducing bleeding events while maintaining efficacy. However, it is important to note that uniform unguided de-escalation may be associated with an increased risk of ischemic events, that would be more difficult to manage than bleeding, since it does not take into consideration the individual patient’s bleeding and ischemic risk in order to select the optimal approach for DAPT abatement, which can lead to serious complications and can be fatal. Further studies will be required to support these assumptions and to determine the most effective approach for individualized patient care.

## Figures and Tables

**Table 1:** Table 1 describes the main characteristics of the de-escalation studies. Abbreviations: BARC: Bleeding Academic Research Consortium Criteria, NACE: net adverse clinical events, ST: stent thrombosis, TIMI: thrombolysis in myocardial infarction, PLATO: platelet inhibition and patient outcomes, MI: myocardial infarction, PRU: P2Y12 reaction unit, SRI: severe recurrent ischemia, CVD: cardiovascular death, UR: urgent revascularization.

Study	TALOS-AMI Trial	HOST-REDUCE-POLYTECH-ACS	TAILOR-PCI	TOPIC	TROPICAL-ACS	-
First author	Park	Kim	Pereira	Cuisset	Sibbing	Ueno
Publication year	2021	2020	2020	2017	2017	2016
Number of patients	2697	2338	5302	646	2610	136
De-escalation strategy	Uniform unguided de-escalation	Uniform unguided de-escalation	Genotype-guided therapy	Uniform unguided de-escalation	Guided by platelet function testing	Uniform unguided de-escalation
Primary outcome	NACE (CVD + MI + Stroke + Bleeding)	NACE (Death + MI + ST + SRI + Bleeding)	CVD + MI + ST + RR + Stroke	CVD + UR + Stroke + Bleeding	CVD + MI + Stroke + Bleeding	PRU
Definition of bleeding (Primary/Secondary)	BARC	BARC	BARC/TIMI	TIMI/BARC	BARC	BARC/TIMI
Treatment used before de-escalation	Ticagrelor + Aspirin	Prasugrel + Aspirin	Ticagrelor + Aspirin	Ticagrelor or Prasugrel + Aspirin	Prasugrel + Aspirin	Prasugrel + Aspirin
Treatment used after de-escalation	Clopidogrel + Aspirin	Prasugrel + Aspirin	Clopidogrel + Aspirin	Clopidogrel + Aspirin	Clopidogrel + Aspirin	Clopidogrel + Aspirin
Clopidogrel (Experimental/Control) (%)	100/0	-	15/99	100/0	100/0	100/0
Prasugrel(Experimental/Control) (%)	0/100	100/100	-	56/59	0/100	0/100
Ticagrelor (Experimental/Control) (%)	0/100	-	85/1	44/42	-	-
Result	Significant decrease in bleeding risk	Reduced risk of NACE	No significant results	Reduced risk of bleeding	No significant results	Increase in PRU

## Data Availability

O. El Alaoui El Abdallaoui had full access to all the data in the study and takes responsibility for the integrity of the data and the accuracy of the data analysis.
